# Interview with Dr. Emma Segura - 8^th^ European Congress on Neurorehabilitation in conjunction with the 20^th^ Congress of the Society for the Study of Neuroprotection and Neuroplasticity

**DOI:** 10.25122/jml-2026-1004

**Published:** 2026-03

**Authors:** Stefana-Andrada Dobran, Alexandra Gherman

**Affiliations:** 1 RoNeuro Institute for Neurological Research and Diagnostic, Cluj-Napoca, Romania; 2 Sociology Department, Babes-Bolyai University, Cluj-Napoca, Romania

**Interviewee: Dr. Emma Segura**

**Interviewer: Ms. Stefana-Andrada Dobran**

Dr. Emma Segura is a postdoctoral researcher at the Cognition and Brain Plasticity Unit of the Bellvitge Biomedical Research Institute in Barcelona, Spain. Her work focuses on developing and evaluating music-based therapies for clinical rehabilitation, exploring how music and brain function interact. She contributed to research in anhedonia and neurorehabilitation for chronic stroke patients, anhedonia in endometriosis, music therapy for chronic pain, and natural singing reward. Currently, she coordinates a major European clinical trial using music therapy for substance use disorder.

**S.D.: Hello, Dr. Emma Segura and welcome to the 8^th^ European Congress of Neurorehabilitation (ECNR) in conjunction with the 20^th^ Congress of the Society for the Study of Neuroprotection and Neuroplasticity. The ECNR brings together the scientific and clinical communities. What do you believe is the unique role it plays in bridging the gap between research and daily patient care in neurorehabilitation?**


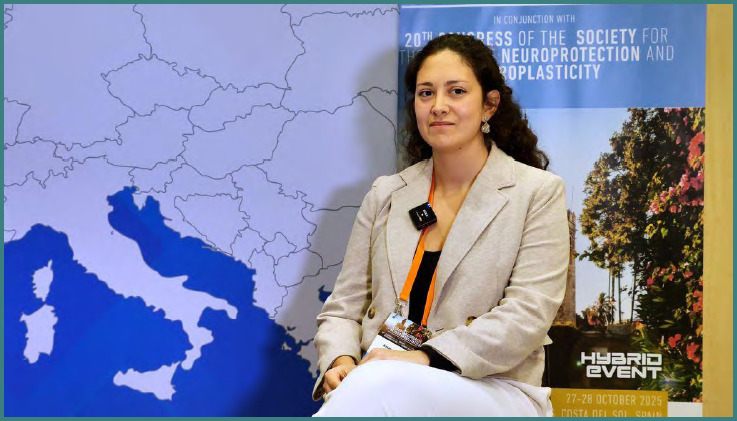


E.S.: Thank you for inviting me. I believe that the ECNR has a unique role in providing truly effective translational research. Scientific research offers insight into the neural mechanisms to develop neurological treatments, while clinical research provides feedback on the feasibility and real-world effectiveness of these treatments and also helps develop the future steps in basic research. So, they provide this bridge–this crucial bridge–to offer the more effective treatments.

**
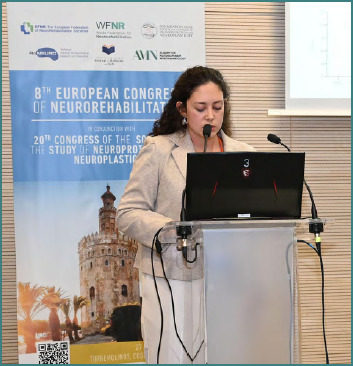
**

**S.D.: Considering your specialty, what future developments do you envision for the complex multidisciplinary field of neurorehabilitation?**

E.S.: I envision a growth in enriched treatments, especially at hospitals, for the early developmental stages of neurological disease to provide a greater multisensory stimulation. This is very effective at the beginning of the disease’s development, and I advise the investment of more resources in these spaces for enriched therapies.

**S.D.: Is there a specific emerging trend or technology that you are excited about in the field?**

E.S.: I think I'm more excited for *augmented reality incorporated in music based interventions*, which is my field. With the advanced technology, we can provide an increased dosage and intensity of our rewarded intervention–the music-based interventions.

**S.D.: In your perspective, what is the most challenging future development in neurorehabilitation and how can the European Federation of NeuroRehabilitation Societies (EFNR) come closer to bridging the gap?**

E.S.: I believe the most challenging [development] is to create robust evidence in general, using more homogeneous measurements, and addressing specific questions, such as [how] to individualize the treatment for each participant–what they need, the specific intensity, time of therapy. All these elements should be addressed more particularly in research, using the same measures, more subjective assessments combined with neuroimaging, and using all this together.

**S.D.: Your presentation discussed how anhedonia can impair motivation for rehabilitation. What are key signs that should not be missed when diagnosing it? In the field of music-supported therapies, what is the most essential element for re-engaging the brain?**

E.S.: Anhedonia is basically a manifestation of a dysfunction of the reward system, so the main symptoms are lack of interest in rewarding experiences such as your hobbies, your pastimes, or socializing with your relatives, friends, and also a decrease in the enjoyment of pleasures such as tasting your favorite meal, listening to your favorite song, for example.


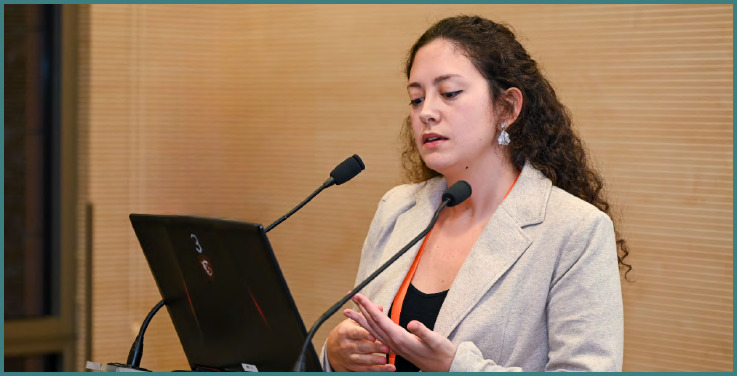


In music-supported therapy, this intervention aims to engage the reward system by providing a meaningful activity that can boost intrinsic motivation of the participants and also increase the sense of urgency and of performing something new that maybe they didn't believe that they would be able to do.

Also, we redesigned this intervention incorporating new motivational aspects adapting the intervention for home use in an autonomous way, incorporating group sessions to increase socialization. This reflects the psychological basic needs of individuals, fulfilling the sense of autonomy, the sense of belonging to a group, and provoking a reward–this is the way of engaging the reward system.

Moreover, a key factor is using the favorite music of participants, as this is the most pleasant stimulus they can work with when training.

